# The relationship between form and function of the carnivore mandible

**DOI:** 10.1002/ar.25678

**Published:** 2025-04-30

**Authors:** Charles J. Salcido, P. David Polly

**Affiliations:** ^1^ Earth & Atmospheric Sciences Indiana University Bloomington Indiana USA

**Keywords:** biomechanics, Carnivora, functional, geometric morphometrics, mandible, morphology

## Abstract

Dietary morphology diversified extensively in Carnivoraformes (living Carnivora and their stem relatives) during the Cenozoic (the last 66 million years) as they evolved to capture, handle, and process new animal and plant diets. We used 3D geometric morphometrics, mechanical advantage, and finite element analysis to test the evolutionary relationship between mandibular form and biomechanical function as subclades independently made the transition from mesocarnivorous diets (50%–70% animal matter) to hypercarnivorous (>70% animal matter) and osteophagous ones (substantial bone processing). We found that mandible shape is correlated with these dietary categories, with mechanical advantage estimates, and with stress and strain caused by the interaction between canine loading and the position of the temporalis relative to the carnassial. The separation of dietary categories is likely related to differences in mandible shape regarding condyle shape, muscle attachment shape, carnassial length, and the length and curvature of the horizontal ramus. This is in turn related to mechanical advantage estimates as the most strongly associated are related to the lengthening of the temporalis lever arm and the shortening of the mandible and the bite point lever arm. The stress and strain differences are likely related to the variation in the distal (or rostral) part of the mandible associated with prey of different sizes (mesocarnivores usually take prey smaller than their own body size, whereas hypercarnivores take prey equal to or larger than themselves). Mesocarnivorous taxa, on average, have higher stress and strain on the mandible than the other diet groups.

## 1 INTRODUCTION

Today, the clade Carnivoraformes, the group of living mammalian carnivores that includes cats, dogs, bears, raccoons, and other taxa and their extinct stem relatives, exhibits a wide variety of dietary ecologies, among which are mesocarnivores (diets consisting of 50%–70% animal matter), hypocarnivores (<50% animal matter), hypercarnivores (>70% animal matter), and bone‐cracking hypercarnivores (Radinsky, 1982; Roemer et al., 2009; Werdelin & Gittleman, 1996). Their variety of diets is accompanied by broad variation in the morphology of their mandibles. Mesocarnivores, like the red fox, *Vulpes vulpes*, and the common genet, *Genetta genetta*, often have long, slender jaws for the quick capture of small prey and the retention of crushing surfaces caudal to the carnassial blade (Friscia et al., 2007; Roemer et al., 2009; Van Valkenburgh, 2007). Hypocarnivores, like the black bear, *Ursus americanus*, and the European badger, *Meles meles*, have jaws with large muscle attachment sites on either the masseteric fossa or the coronoid process, small shearing surfaces on their carnassial, and a greater crushing area of the molars behind the carnassial shearing surface (Greaves, 1983; Meloro & Raia, 2010; Van Valkenburgh, 2007). Hypercarnivores, like the tiger, *Panthera tigris*, and the long‐tailed weasel, *Mustela frenata*, have short jaws to reduce the out‐lever of the bite force, longer coronoid processes and larger condyles, canines, mandibular symphysis, and carnassial shearing surface (Van Valkenburgh, 1991, 1999, 2007; Van Valkenburgh et al., 2004). Bone‐cracking hypercarnivores, like the spotted hyaena, *Crocuta crocuta*, possess more robust teeth and a dorsoventral displacement of the ascending ramus and the horizontal ramus of the mandible (Ferretti, 2007; Tseng, 2013; Tseng et al., 2011; Tseng & Wang, 2010; Van Valkenburgh, 1999, 2007). These morphologies vary in their functional performance as measured by independent metrics like mechanical advantage or total stress and strain.

**FIGURE 1:**
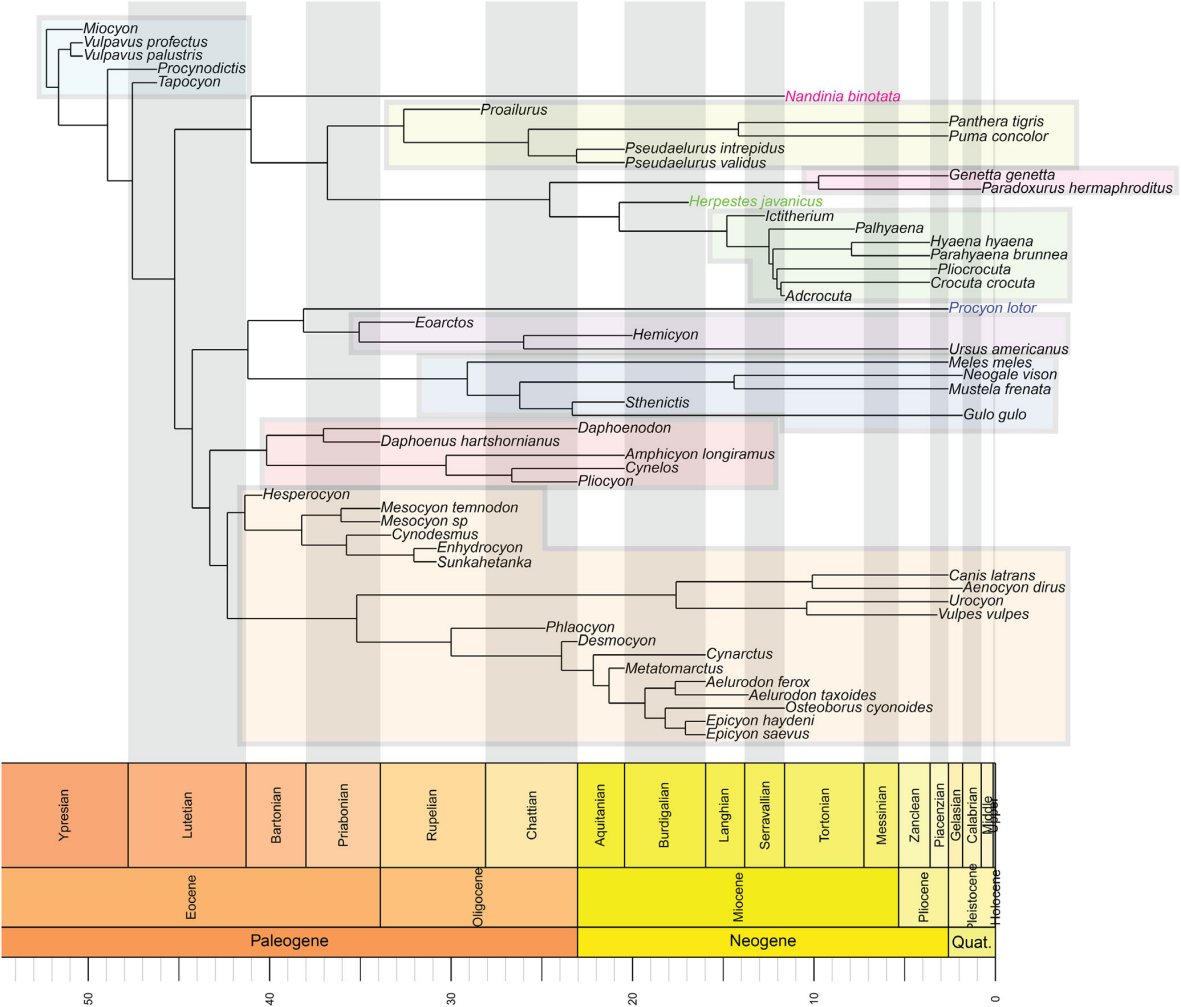
Time‐calibrated phylogeny of Carnivoraformes with major clades of interest highlighted. Light blue, “Miacidae”; Yellow, Felidae; Pink, Viverridae/civets; Green, Herpestidae + Hyaenidae; Dark blue, Procyonidae; Purple, Ursidae; Light blue, Mustelidae; Red, Amphicyonidae; Orange, Canidae. Tree made from Wozencraft (1989), Baskin (1998), Flynn et al. (2005), and Spaulding and Flynn (2012). Dating based on first and last appearance dates from the Paleobiology Database (Peters & McClenn, 2016).

Carnivoraformes is comprised of several extant subclades and a host of extinct taxa that are closely related to them (Figure 1; Baskin, 1998; Flynn et al., 2005; Spaulding & Flynn, 2012; Wozencraft, 1989). The paraphyletic group Miacidae, which are the earliest members of the group and are closely related to the extant groups, had a variety of mesocarnivorous specializations and most of which retained the plesiomorphic dental plan of Carnivoraformes (I3/3, C1/1, P4/4, M3/3). The genus *Vulpavus* represents the miacids in this study (Radinsky, 1982; Solé et al., 2016; Sole & Smith, 2013; Spaulding & Flynn, 2012). The small range in body size and the ubiquitous mesocarnivorous diet in early carnivoriforms has been attributed to constraints imposed by competitive exclusion from other phylogenetically independent and now‐extinct mammalian carnivore groups, including Mesonychia, Hyaenodonta, and Oxyaenodonta (Martin, 1989; Van Valkenburgh, 1988, 1999, 2007). By the late Eocene, around 42 million years ago, members of the crown group Carnivora began diverging into what would become Canidae, Mustelidae, early groups of Feliformia, and extinct groups such as Amphicyonidae. By the Miocene epoch 20 million years ago, all extant Carnivora families had evolved, including Felidae, Hyaenidae, Herpestidae, Ursidae, Procyonidae, and Viverridae (Gregory & Hellman, 1939; Hunt Jr, 1998; Martin, 1989; Van Valkenburgh, 1991, 1999; Wozencraft, 1989).

Generalist mesocarnivory was the ancestral dietary ecology of Carnivoraformes, and the other dietary specializations (hypocarnivores, hypercarnivores, and bone‐cracking hypercarnivores) evolved as the clade diversified (Greaves, 1985; Martin, 1989; Radinsky, 1982; Van Valkenburgh, 2007). Certain differences in mandible shape have been inferred to be the functional consequence of these specializations, and if so, we would expect phylogenetic changes in shape to be associated with changes in biomechanical performance (Davis, 2014; Dullemeijer & Barel, 1977; Greaves, 1985; Heinrich, 1996; Herring, 1993; Meloro et al., 2015; Radinsky, 1982; Werdelin & Gittleman, 1996). Because hypocarnivory, hypercarnivory, and bone cracking specializations have evolved independently several times in different carnivoraform subclades, we expect that mandible shape should show ecomorphological convergence (statistically significant “ecomorphs”) (Christiansen & Adolfssen, 2005; Goswami & Polly, 2010; Meloro & Raia, 2010; Van Valkenburgh, 2007). For example, some authors argue that a hypercarnivorous “felid” ecomorph that is today found not only in cats but which is also found in extinct caniforms like the mustelid *Megalictis* and canid *Enhydrocyon* whose cat‐like morphologies have been proposed to be the result of them filling cat‐like ecological roles during the North American “cat gap” (27–17 Ma) (Christiansen, 2008; Goswami et al., 2011; Meloro et al., 2015; Van Valkenburgh, 1991, 1999, 2007). Another example is the osteophagous “hyaenid” ecomorph found in Hyaenidae and some Canidae (specifically the subfamily Borophaginae) that consists of a domed cranium, an ascending ramus that is displaced from the horizontal ramus, a large, vertically aligned coronoid process, and more robust dentition (Ferretti, 2007; Tseng, 2013; Tseng et al., 2011; Van Valkenburgh, 2007). If these shape ecomorphs are truly optimized for the biomechanical requirements of these respective dietary strategies, then we expect to see correlated changes in mandible shape and biomechanical function as different subclades have evolved these specializations.

In this paper we test these propositions using three‐dimensional (3D) digital scans of carnivoraform mandibles sampled from several independent transitions from the ancestral mesocarnivorous diet to more derived ones to test the connection between diet, mandibular shape, and functional performance. We hypothesize that phylogeny will have a strong correlation to the variation of mandible shape, but that diet and functional performance that are associated with ecomorphs will have an even stronger correlation that transcends phylogenetically specific aspects of form. We will use finite element analysis, mechanical advantage measurements, and geometric morphometric analysis to document the patterns of bite force and mandibular stress and strain evolution in the carnivoraform mandible. These patterns will be analyzed in a phylogenetic context to determine whether transitions between mesocarnivory and hypercarnivory are universally associated with a shift from high‐stress mandible shape to lower stress forms. Hypocarnivorous taxa were also included in this study to provide a contrast to the hypercarnivorous and bone‐cracking taxa.

## 2 MATERIALS AND METHODS

### 2.1 Institution abbreviations and dental terminology

Institutional abbreviations: The American Museum of Natural History (AMNH), Indiana University William R. Adams Zooarchaeology Collections (WRAZL), University of Michigan Museum of Zoology (UMMZ), the University of Florida's Florida Museum of Natural History (UF), Museum national d'Histoire naturelle (MNHN), Hefner Museum of Natural History (MUZO), University of California Berkeley Museum of Vertebrate Zoology (MVZ), Idaho Museum of Natural History (IMNH), North Carolina State University (NCSU), Musem van Naturlijke Historie, Leiden (MNHL), the Smithsonian Institution National Museum of Natural History (USNM), University of Arkansas Museum (UAM), San Diego Museum of Natural History (SDMNH), and the Yale Peabody Museum (YPM).

Dental terminology and abbreviations generally follow Bown and Krause (1979). Here the following tooth type abbreviations are used, with lowercase indicating a mandibular tooth and uppercase indicating a maxillary one: i, incisor; c, canine; p, premolar; m, molar.

### 2.2 Specimens

A total of 54 specimens were chosen to sample as many putative phylogenetic transitions from mesocarnivory to one of the specialized dietary ecologies as possible, of which 17 are extant and the remainder are extinct. Each species is represented by a single adult mandible as determined by having full permanent dentition. Fossil mandibles are often missing the condyle, coronoid process, or key teeth, and in some cases, our sampling of fossil taxa was constrained by the availability of complete or near complete mandibles that were not taphonomically deformed. The taxonomic breakdown can be seen in Table 1. Our selection includes 80% of the 15 extant and extinct families of Carnivoraformes, with only Ailuridae (red pandas, which are dominantly omnivorous), Eupleridae (an exclusively Malagasy radiation), and Prionodontidae (the linsangs) missing (Martin, 1989; Van Valkenburgh, 2007).

**TABLE 1:** List of all specimens used in this study along with taxonomic data, specimen data, and source of mesh data.

Taxa	Larger cladistic groups	Family	Ecology	Collection	Catalog no.	Period	Data type	Source
Adcrocuta	Carnivoramorpha	Hyaenidae	Bone‐cracker	AMNH	22,880	Miocene	Photogrammetry	Database and collections visit
*Aelurodon ferox*	Carnivoramorpha	Canidae	Bone‐cracker	USNM	352,364	Miocene	Photogrammetry	Collections visit
*Aelurodon taxoides*	Carnivoramorpha	Canidae	Bone‐cracker	AMNH	67,008	Miocene	Photogrammetry	Collections visit
*Aelurodon taxoides*	Caniformia	Canidae	Bone‐cracker	YPM	VPPU 10635	Miocene	Photograph	Collections visit
*Aenocyon dirus*	Carnivoramorpha	Canidae	Hypercarnivore	YPM	VPPU 017990B	Pleistocene	Surface scanned	Shared
*Amphicyon longiramus*	Carnivoramorpha	Amphicyonidae	Bone‐cracker	UF	VP 019909	Miocene	Surface scanned and photograph	Collections visit
*Canis latrans*	Carnivoramorpha	Canidae	Mesocarnivore	UAM	88–50‐290	Holocene	CT‐scanned	Morphosource
*Crocuta crocuta*	Carnivoramorpha	Hyaenidae	Bone‐cracker	MVZ	165,169	Holocene	Surface scanned	Morphosource
Cynarctus	Carnivoramorpha	Canidae	Hypocarnivore	USNM	3,524,050	Miocene	Photogrammetry	Collections visit
Cynelos	Carnivoramorpha	Amphicyonidae	Mesocarnivore	UF	VP 211624	Miocene	Surface scanned and photograph	Collections visit
Cynodesmus	Carnivoramorpha	Canidae	Mesocarnivore	YPM	VPPU 010412	Oligocene	Surface scanned	Shared
Daphoenodon sp	Carnivoramorpha	Amphicyonidae	Bone‐cracker	UF	VP 270828	Miocene	Surface Scanned and Photograph	Collections Visit
*Daphoenus hartshornianus*	Carnivoramorpha	Amphicyonidae	Mesocarnivore	UF	VP 207948	Oligocene	Surface scanned and photograph	Collections visit
Desmocyon	Carnivoramorpha	Canidae	Mesocarnivore	AMNH	FM 49177	Oligocene	Photogrammetry	Collections visit and database
Enhydrocyon	Carnivoramorpha	Canidae	Hypercarnivore	AMNH	12,886	Oligocene	Photogrammetry	Collections visit
Eoarctos	Carnivoramorpha	Ursidae	Hypocarnivore	USNM	PAL 637259	Oligocene	Laser scan	Morphosource
*Epicyon haydeni*	Carnivoramorpha	Canidae	Bone‐cracker	AMNH	61,462	Miocene	Photogrammetry	Collections visit
*Epicyon saevus*	Carnivoramorpha	Canidae	Bone‐cracker	UF	VP 37265	Miocene	Surface scanned and photograph	Collections visit
*Genetta genetta*	Carnivoramorpha	Viverridae	Mesocarnivore	MNHN	ZM MO‐1997‐450		Surface scanned	Morphosource
*Gulo gulo*	Carnivoramorpha	Mustelidae	Bone‐cracker	AMNH	M‐182936	Holocene	CT‐scanned	Morphosource
Hemicyon	Carnivoramorpha	Ursidae	Hypercarnivore	UF	VP 19908	Miocene	Surface scanned and photograph	Collections visit
*Herpestes javanicus*	Carnivoramorpha	Herpestidae	Mesocarnivore	USNM	293,772	Holocene	CT‐scanned	Morphosource
Hesperocyon	Carnivoramorpha	Canidae	Mesocarnivore	USNM	PAL 336368	Oligocene	Photogrammetry	Collections visit and database
*Hyaena hyaena*	Carnivoramorpha	Hyaenidae	Bone‐cracker	USNM	182,034	Holocene	CT‐scanned	Morphosource
Ictitherium	Carnivoramorpha	Hyaenidae	Mesocarnivore	AMNH	FAM 144906	Miocene	Photogrammetry	Collections visit
*Meles meles*	Carnivoramorpha	Mustelidae	Hypocarnivore	AMNH	M‐70603	Holocene	CT‐scanned	Morphosource
Mesocyon sp.	Carnivoramorpha	Canidae	Mesocarnivore	USNM	V 7916	Oligocene	Photogrammetry	Collections visit and database
Mesocyon temnodon	Carnivoramorpha	Canidae	Mesocarnivore	USNM	FM 63367	Oligocene	Photogrammetry	Collections visit
Metatomarctus	Carnivoramorpha	Canidae	Mesocarnivore	UF	VP 256998	Miocene	Surface scanned and photograph	Collections visit and database
Miocyon	Carnivoramorpha	Miacidae	Mesocarnivore	SDNHM	40,814	Eocene	Surface scanned	Shared
*Mustela frenata*	Carnivoramorpha	Mustelidae	Hypercarnivore	USNM	95,054	Holocene	Surface scanned	Morphosource
*Nandinia binotata*	Carnivoramorpha	Viverridae	Hypocarnivore	USNM	450,440	Holocene	CT‐scanned	Morphosource
*Neogale vison*	Carnivoramorpha	Mustelidae	Hypercarnivore	IMNH	R 103	Holocene	Laser scan	Morphosource
*Osteoborus cyonoides*	Carnivoramorpha	Canidae	Bone‐cracker	AMNH	FM 61640	Miocene	Photogrammetry	Collections visit
Palhyaena	Carnivoramorpha	Hyaenidae	Bone‐cracker	AMNH	FAM 144897	Miocene	Photogrammetry	Collections visit
*Panthera tigris*	Carnivoramorpha	Felidae	Hypercarnivore	WRAZL	8,610,108	Holocene	Photogrammetry	Collection's visit
*Paradoxurus hermaphroditus*	Carnivoramorpha	Viverridae	Hypocarnivore	MNHN	ZM AC‐A3448	Holocene	Surface scanned	Morphosource
*Parahyaena brunnea*	Carnivoramorpha	Hyaenidae	Bone‐cracker	MVZ	117,842	Holocene	CT‐scanned	Morphosource
Phlaocyon	Carnivoramorpha	Canidae	Hypocarnivore	AMNH	FM 8768	Oligocene	Photogrammetry	Collections visit
Pliocrocuta	Carnivoramorpha	Hyaenidae	Bone‐cracker	MNHL	20,161,768	Pliocene	Surface scanned	Shared
Pliocyon	Carnivoramorpha	Amphicyonidae	Bone‐cracker	UF	VP 24013	Miocene	Surface scanned and photograph	Collections visit and database
Proailurus	Carnivoramorpha	Felidae	Hypercarnivore	AMNH	101,931	Miocene	Photogrammetry	Collections visit and database
Procynodictis	Carnivoramorpha	Miacidae	Mesocarnivore	SDNHM	42,810	Eocene	Surface scanned	Shared
*Procyon lotor*	Caniformia	Procyonidae	Hypocarnivore	UMMZ	98,905	Holocene	Photograph	Database
*Pseudaelurus intrepidus*	Carnivoramorpha	Felidae	Hypercarnivore	AMNH	V 124	Miocene	Photogrammetry	Collections visit
*Pseudaelurus validus*	Carnivoramorpha	Felidae	Hypercarnivore	AMNH	62,128	Miocene	Photogrammetry	Collections visit and database
*Puma concolor*	Carnivoramorpha	Felidae	Hypercarnivore	WRAZL		Holocene	Photogrammetry	Collections visit
Sthenictis	Carnivoramorpha	Mustelidae	Hypercarnivore	AMNH	FM 25235	Miocene	Photogrammetry	Collections visit and database
Sunkahetanka	Carnivoramorpha	Canidae	Hypercarnivore	YPM	VPPU 013602	Oligocene	Surface scanned and photograph	Shared and collections visit
Tapocyon	Carnivoramorpha	Miacidae	Mesocarnivore	SDNHM	36,000	Eocene	Surface scanned	Shared
Urocyon	Carnivoramorpha	Canidae	Mesocarnivore	MUZO	MT 1117	Holocene	Surface scanned	Morphosource
*Ursus americanus*	Carnivoramorpha	Ursidae	Hypocarnivore	NCSU	371,168	Holocene	Structured light	Morphosource
*Vulpavus palustris*	Carnivoramorpha	Miacidae	Mesocarnivore	AMNH	19,000	Eocene	Photogrammetry	Collections visit
*Vulpavus profectus*	Carnivoramorpha	Miacidae	Mesocarnivore	USNM	PAL 362805	Eocene	Photogrammetry	Collections visit and database
*Vulpes vulpes*	Carnivoramorpha	Canidae	Mesocarnivore	UCLA	Mammals 15,180	Holocene	CT‐scanned	Morphosource

We produced 3D digital mesh models from our own original scans using photogrammetry, surface scanning, or CT‐scanning and downloaded additional meshes from the Morphosource repository (Boyer et al., 2016). Table 1 reports the taxa, mesh sources, and specimen data for the specimens in our study. Photogrammetry is the method in which 3D information is extracted from photographs to create a 3D model (Mikhail et al., 2001). Photographs were taken via a Nikon D5600 DSLR camera with either a 18–55 mm lens or an AF‐S DX Micro NIKKOR 85 mm f 3 5G ED VR lens (depending on size of the specimen) on a manual turntable in a LED lightbox with diffused lighting. Agisoft Metashape 1.8.5 (Agisoft LLC) was the software used to create digital models from the photographs taken by the camera in the photogrammetry procedure. Surface scanning was done via either a NextEngine 3D Scanner or an Arctec Spyder Scanner depending on what was available at the institution in which the samples were surveyed. CT‐scanning was done on specimens that were typically downloaded from the online three‐dimensional data repository Morphosource and segmented into a 3D mesh via the software 3D Slicer (Pieper et al., 2004). The procedure used to acquire the 3D meshes has no consequence for the study, only on the resolution and quality of the output surface model for the specimen from which we identified homologous features on which to place landmarks.

### 2.3 Geometric morphometrics

**FIGURE 2:**
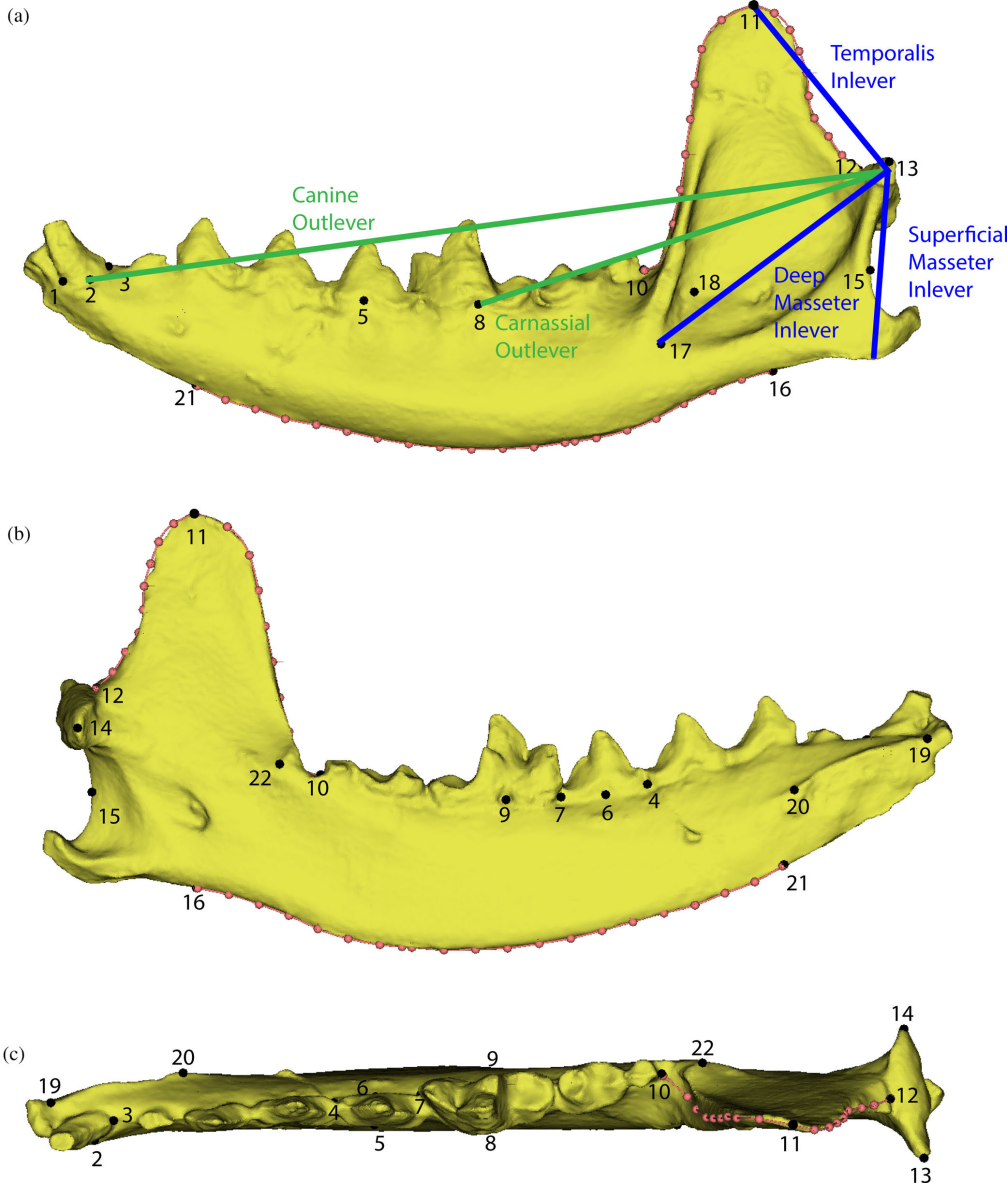
Landmarking scheme for geometric morphometrics analysis using *Hesperocyon* as an example. Fixed landmarks are red and semilandmarks are purple.

Geometric morphometrics is the analysis of shape using Cartesian landmark and semilandmark coordinates (Adams et al., 2004; Bookstein, 1991; Dryde & Mardia, 1998; Perez et al., 2006). 3D coordinates were collected from digital models using the markup tool in the software 3D Slicer (Pieper et al., 2004). Landmarks were collected from either the left mandible or a mirrored right mandible when the former was unavailable or incomplete. Twenty‐two fixed landmarks were taken from the mandible as outlined in Figure 2. These landmarks were derived based on Meloro et al. (2015), Meloro and O'Higgins (2011), and Prevosti et al. (2012) to highlight key areas associated with diet and function.

Semilandmark curves were also used to create evenly spaced points that trace the shape of the mandible's edge in three functionally important regions: one curve to capture the rostral curve of the coronoid process between landmarks 10 and 11 with 10 evenly spaced points, one curve to capture the caudal curve of the coronoid process between landmarks 11 and 12 with 10 evenly spaced points, and one curve to capture the ventral side of the horizontal ramus of the mandible between landmarks 16 and 21 with 20 evenly spaced points. In total, the 3D models have 62 landmarks (22 fixed landmarks and 40 semilandmarks) (Figure 2).

Geometric morphometric analysis was carried out in R (Ihaka & Gentleman, 1996) using *rStudio* with the R package *geomorph* v4.0.5 (Adams & Otárola‐Castillo, 2013). Landmarks were Procrustes superimposed and submitted to principal component analysis (PCA) of shape to visualize what groups of landmarks drive variation in shape. A permutation‐based Procrustes analysis of variance (ANOVA; Klingenberg & McIntyre, 1998) was used to test the association of shape with functional‐morphological and biomechanical metrics of mechanical advantage (MA) of the temporalis and masseteric bites (measured separately at the canine and carnassial), the mesh‐weighted arithmetic mean (MWAM) Von Mises stress and strain when biting on the canine and carnassial separately (estimated with finite element analysis, as described below), and a single‐factor multivariate analysis of covariance (MANCOVA; Adams & Collyer, 2018; Huberty & Petoskey, 2000) of ecological categories and taxonomic family‐level groupings. To assess the effect of phylogeny, a phylogenetic generalized least square (PGLS) was also used to test the association of shape with functional morphological and biomechanical metrics. This was paired with a generalized K‐statistic representing the degree of phylogenetic signal. A time‐calibrated phylogenetic tree was made from Baskin (1998), Wozencraft (1989), Flynn et al. (2005), and Spaulding and Flynn (2012). Dating was based on first and last appearance dates from the Paleobiology Database (Peters & McClenn, 2016).

Mechanical advantage was taken as a proxy for bite force. Mechanical advantage is the ratio of the length of the in‐lever (the lever arm producing force, i.e., the muscle) to the length of the out‐lever (the lever arm where the force of the in‐lever is transmitted to, i.e., the bite point). Regression of metrics such as mechanical advantage onto Procrustes shape is to extract the part of the mandible that is directly related to lever arms.

### 2.4 Finite element analysis

Finite element analysis (FEA) was applied to the digital models to calculate the stress and strain imposed by simulated bites at two locations along the tooth row. Finite element analysis simulates how an object behaves when force(s) is (are) applied by treating the model as many discrete, connected elements (Korioth et al., 1992; Rayfield, 2007; Richmond et al., 2005; Ross, 2005). FEA solves a stiffness matrix where forces are the inputs and displacements (or deformation in every node), and then, via an integration process in the elements, stress and strain are obtained.

Before performing FEA, the models were edited in Geomagic (3D Systems Corp; Hai, 2007) to remove the crowns of the teeth because of different tooth counts and, especially in the case of fossil specimens, missing teeth (Gill et al., 2014; Morales‐García et al., 2019). The mandible and remaining tooth roots were integrated as one body as they are both stiff and this integration has been used by other authors (Gill et al., 2014; Morales‐García et al., 2019; Tseng, 2013). Digital models of fossil specimens were also prepared for FEA following the recommendations described in Lautenschlager (2016) by fixing cracks and filling holes using Geomagic and Blender (Brito, 2007). Once the models were prepped, a convergence test on element resolution determined the minimum number of elements necessary to reproduce the same stress and strain results as higher resolution models (Bright & Gröning, 2011; Jones & Wilcox, 2008; Maas et al., 2012). The minimum number of nodes, faces, and elements was found on average to be 143,000, 223,000, and 514,000 respectively based on using tetrahedral elements for the mesh.

The model elements were assigned FEA isotropic elastic properties with a Young's modulus of 20 GPa and a Poisson ratio of 0.3 based on the average properties of mammalian cortical bone (Currey, 1984; Dumont et al., 2005) and previous FEA studies on Carnivora skulls (Tseng, 2009; Tseng et al., 2011; Tseng & Flynn, 2015). The whole mandible was assigned these properties, ignoring the potential roots of the dentition, as FEA will determine how the different shapes of the mandible will handle different force loads and distribute stress (Gill et al., 2014; Morales‐García et al., 2019; Porro et al., 2011).

**FIGURE 3:**
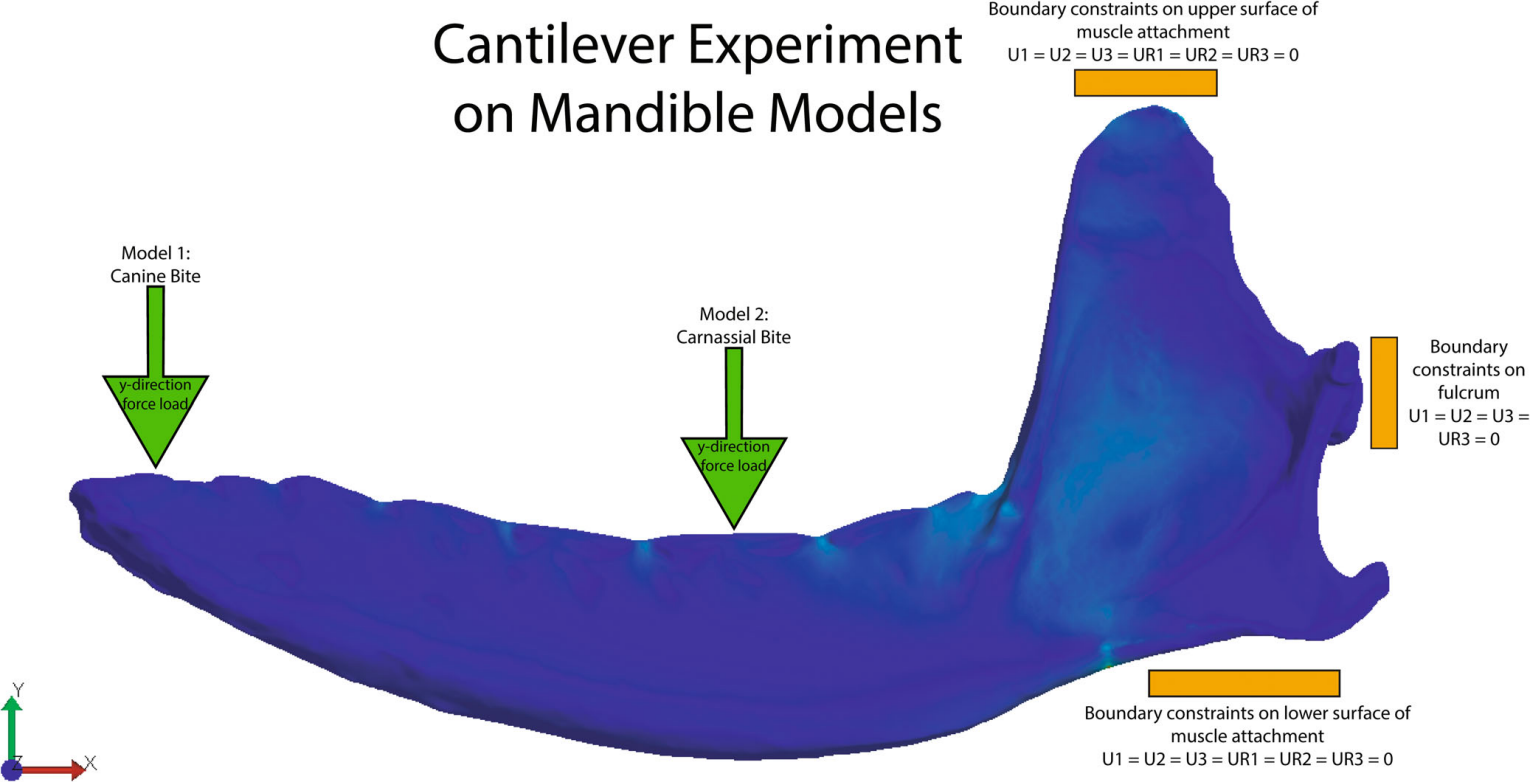
Free‐body diagram of finite element analysis model loading with *Hesperocyon* as example. Constraints are marked in red and loading points are marked in green.

FEA boundary constraints were placed on the mandibular condyle and the most dorsal and ventral areas of muscle attachment to create a counter‐lever experiment (Tseng et al., 2023). Multipoint constraints were placed on these areas with four defined degrees of freedom (for dorsal and ventral muscle attachment areas: U1 = U2 = U3 = UR1 = UR2 = UR3 = 0; for the mandibular condyle: U1 = U2 = U3 = UR3 = 0; U1 is the mediodistal axis, U2 is the dorsovental axis, and U3 is the axis along the jaw length; U describes translational movement and UR describes rotational movement) (Gill et al., 2014). The mandibular condyle constraints allow only for the expected movement of the joint as it would in vivo (Figure 3; orange rectangles).

We used two models, one where an extrinsic force load was placed at the canine and another where the extrinsic force load was placed on the carnassial (Figure 3; green triangles). These locations have different leverages (rostral versus caudal) and different functions (acquiring/subduing prey with the canine versus chewing/processing food with the carnassial/molar area). *Vulpavus profectus*, a small, basal carnivore and inferred mesocarnivore, was loaded with 100 N at each tooth area depending on the model (canine or carnassial). This 100 N load on *Vulpavus* was then scaled according to the volume ratio to the 2/3rds power of other mandibles to produce a comparable load for other models and allow a comparison of stress and strain on the mandible based on differences in mandibular shape (Dumont et al., 2005; Fortuny et al., 2015; Marcé‐Nogué et al., 2013; Rayfield, 2007). The MWAM of the Von Mises stress and the effective strain were recorded from each bite point of each model (Marcé‐Nogué et al., 2017; Serrano‐Fochs et al., 2015).

## 3 RESULTS

### 3.1 Geometric morphometric analysis

**FIGURE 4:**
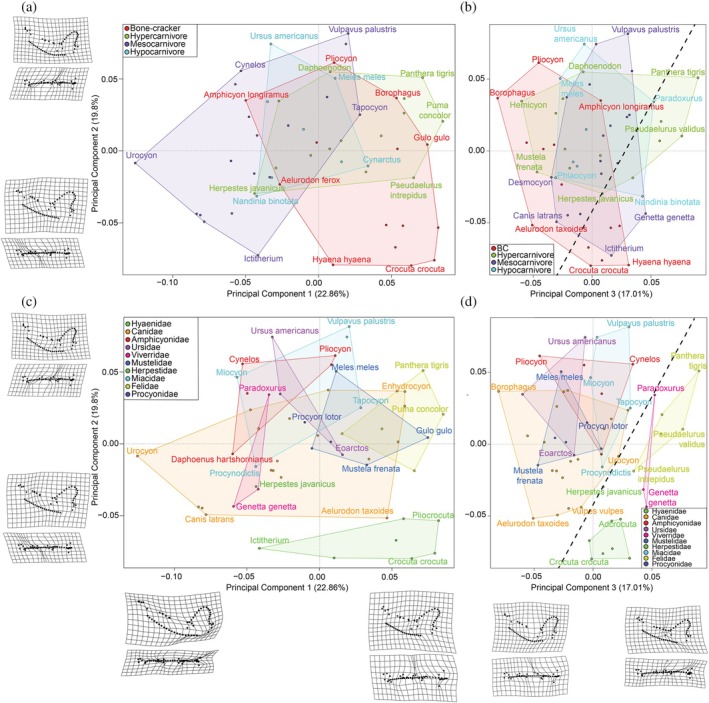
Principal components of shape for Carnivoraformes jaws. Groupings are ecology (a and b) and phylogeny (c and d). Principal components compared include PC1 versus PC2 (a and c) and PC3 versus PC2 (b and d), keeping PC2 on the *x*‐axis. Dashed lines in c and d separate Caniformia and Feliformia.

Mandible shape differences that have previously been inferred to relate to biomechanical function include the ventral curvature of the mandible, the orientation and thickness of the coronoid process, the thickness of the ramus, and the width of the condyle (Greaves, 1985; Van Valkenburgh, 1991, 1999, 2007; Van Valkenburgh et al., 2004). To assess the dietary and phylogenetic patterning in mandibular shape differences, the first three axes (of 53 total axes) of a PCA morphospace, which comprises approximately 54% of the total shape variance, were constructed with each ecological or phylogenetic group delimited with a convex hull (Figure 4).

PC1 represents 24.96% of total shape variance. The positive scores of PC1 represent a relatively high but narrow condyle, a short carnassial blade relative to a longer crushing area of the molars, a relatively long rostrum, a relatively small masseteric fossa, a relatively short coronoid process, a more curved ventral body of the mandible, and a relatively long, horizontal mandibular symphysis. The most extreme taxon on these scores is *Urocyon* (the gray fox). The negative scores of PC1 represent a relatively wide, rostrally placed condyle, a long carnassial blade relative to a smaller crushing area of the molars, a relatively short rostrum, a relatively rostral masseteric fossa, a relatively large, distally placed coronoid process, a comparatively straight ventral body of the mandible, and a relatively large, vertical mandibular symphysis with a large canine. The most extreme taxon on these scores is *Puma* (the mountain lion).

PC2 represents 17.32% of total shape variance. The negative scores of PC2 are a long carnassial blade, a more caudal fourth premolar, a more rostral mandibular symphysis, and a more ventral masseteric fossa; the most extreme taxon is *Crocuta* (the spotted hyena). The positive scores of PC2 represent a more rostral fourth premolar and carnassial blade, a more caudal mandibular symphysis; the most extreme taxon is *Vulpavus*.

PC3 represents 12.19% of total shape variance. The negative scores of PC3 represent a more angled horizontal ramus, a higher, steeper coronoid process, a more rostral fourth premolar and carnassial, a more rostral masseter, and a steep coronoid process; the most extreme taxon is *Borophagus*. The positive scores of PC3 are a straighter horizontal ramus, a smaller crushing area of the molars, a lower, less steep coronoid process, and a more rostral masseter; the most extreme taxon is *Panthera* (specifically the tiger).

The first PC appears to be a dietary contrast between mesocarnivores and the specialized hypercarnivorous and bone‐cracking morphologies, with some overlap in the middle (Figure 4). There is little separation between the dietary categories apparent on PC2 and PC3. Mesocarnivores occupy a larger part of the morphospace than do the other dietary categories, with bone‐crackers having the second largest dispersion, followed by other hypercarnivores. The overlap of bone‐crackers and mesocarnivores is largely driven by amphicyonids (an extinct group of canid‐like carnivores colloquially referred to as “bear‐dogs”) where some of the species have been inferred to be bone‐crackers (e.g., *Amphicyon*). Hypocarnivores appear in the center between the other three groups. Hypocarnivores are nested generally in the middle of all other groups, where they overlap with each other, mostly to the negative of PC1, positive end of PC2, and centered on PC3. There is a higher overlap between mesocarnivores and hypocarnivores when looking at PC2 and PC3 (Figure 4b).

Phylogenetic groupings are most apparent on PC2 and PC3 (Figure 4d) where Hyaenidae, Felidae, and Viverridae are shown. Canidae occupies the largest area in the plots, and the Caninae (i.e., *Canis*, *Vulpes*, *Urocyon*, and *Aenocyon*) are distinct from other canids (i.e., the Hersperocyoninae such as *Hesperocyon* and Borophaginae such as *Epicyon*). A combination of PC2 and PC3 shows a separation between Feliformia and Caniformia (Figure 4b,d). For the first PCs, the two species of the genus *Vulpavus*, a more basal genus of carnivore, are usually distinct from the rest of the carnivores, whereas *Tapocyon* aligns closely with mustelids and canids, *Miocyon* with amphicyonids, and *Procynodictis* with canids and viverrids. It should be noted that *Herpestes* is on the line that separates Feliformia and Caniformia. A larger sampling of Herpestidae or other smaller or morphologically more generalized in appearance feliforms, such as Viverridae and Eupleridae, might make this separation less apparent on these PCs.

### 3.2 Functional morphology: mechanical advantage

The mechanical advantage estimates were regressed onto Procrustes distances of the respective taxa to determine the explanatory power that each function metric has on mandible shape (Table 2). *R* ^2^ values were no greater than 0.14 for any metric, with an average of 0.10, and all were shown to be significant (*p*‐value <0.05). The highest value was of the mechanical advantage of the temporalis and the canine (*R* ^2^ = 0.20, *p*‐value = 0.001) and the lowest value was the mechanical advantage of the superficial masseter and the canine (*R* ^2^ = 0.06, *p*‐value = 0.004). The explanatory power for most of the metrics is improved under a MANCOVA when phylogeny is covarying with shape.

**TABLE 2:** List of *p*‐values and *R* ^2^ values of the respective categorical and functional metrics from Procrustes ANOVA and phylogenetic least squares (PGLS) and the most associated principal components from regression.

Category or functional metric	*p*‐Value of category or metric onto Procrustes shape	*R* ^2^ of category or metric onto Procrustes shape	Three highest correlated principal components of shape onto metric	*p*‐Value of category or metric onto Procrustes shape (PGLS)	*R* ^2^ of category or metric onto Procrustes shape (PGLS)
Ecology	0.001	0.18	NA	0.168	0.09
Family	0.001	0.38	NA	0.707	0.18
Suborder	0.001	0.14	NA	0.233	0.09
MA Canine—Temporalis	0.001	0.2	1,4,7	0.031	0.06
MA Carnassial—Temporalis	0.001	0.14	1,2,4	0.044	0.05
MA Canine—Superficial Masseter	0.004	0.056	1,7,4	0.109	0.05
MA Carnassial—Superficial Masseter	0.001	0.08	1,7,4	0.158	0.05
MA Canine—Deep Masseter	0.001	0.08	1,3,2	0.050	0.02
MA Carnassial—Deep Masseter	0.001	0.10	1,3,2	0.081	0.02
FEA—stress at Canine	0.001	0.15	1,5,4	0.035	0.05
FEA—strain at Canine	0.001	0.15	1,5,4	0.035	0.05
FEA—stress at Carnassial	0.001	0.09	1,2,6	0.068	0.07
FEA—strain at Carnassial	0.001	0.08	1,2,6	0.068	0.06

Abbreviation: MA, mechanical advantage.

For the PGLS, all metrics showed the influence of phylogeny in their correlation with shape as all *R* ^2^ values decreased and some became insignificant (*p*‐value >0.05). The only metrics that still retained significant values were the mechanical advantages of the temporalis and the canine (*R* ^2^ = 0.06, *p*‐value = 0.031), the temporalis and the carnassial (*R* ^2^ = 0.05, *p*‐value = 0.044), and the deep masseter and the canine (*R* ^2^ = 0.02, *p*‐value = 0.05). A generalized *K*‐statistic on the degree of phylogenetic signal on shape variables was 0.3276, showing some effect of phylogeny on mandible shape.

When principal component scores were regressed onto functional metrics, nearly all the metrics are most correlated with PC1 (Table 2). PC2 is highly correlated with the mechanical advantage of the temporals at the carnassial and both of the deep masseter measurements, and carnassial stress. PC3 is only highly correlated with the deep masseter mechanical advantage metrics (Table 2). Lower PCs that are also more highly correlated with the metrics include PC4 (most mechanical advantage measurements except with the deep masseter), PC5 (stress and strain of the canine), PC6 (stress and strain of the carnassial), and PC7 (mechanical advantage of the temporalis and the canine and both superficial masseter measurements). PC4, PC5, PC6, and PC7 represent 9.22%, 6.2%, 4.27%, and 3.53% of total shape variance in that order and totaling 23.22% of total shape variance (with PCs 1–3 making approximately 77% of total shape variance).

**FIGURE 5:**
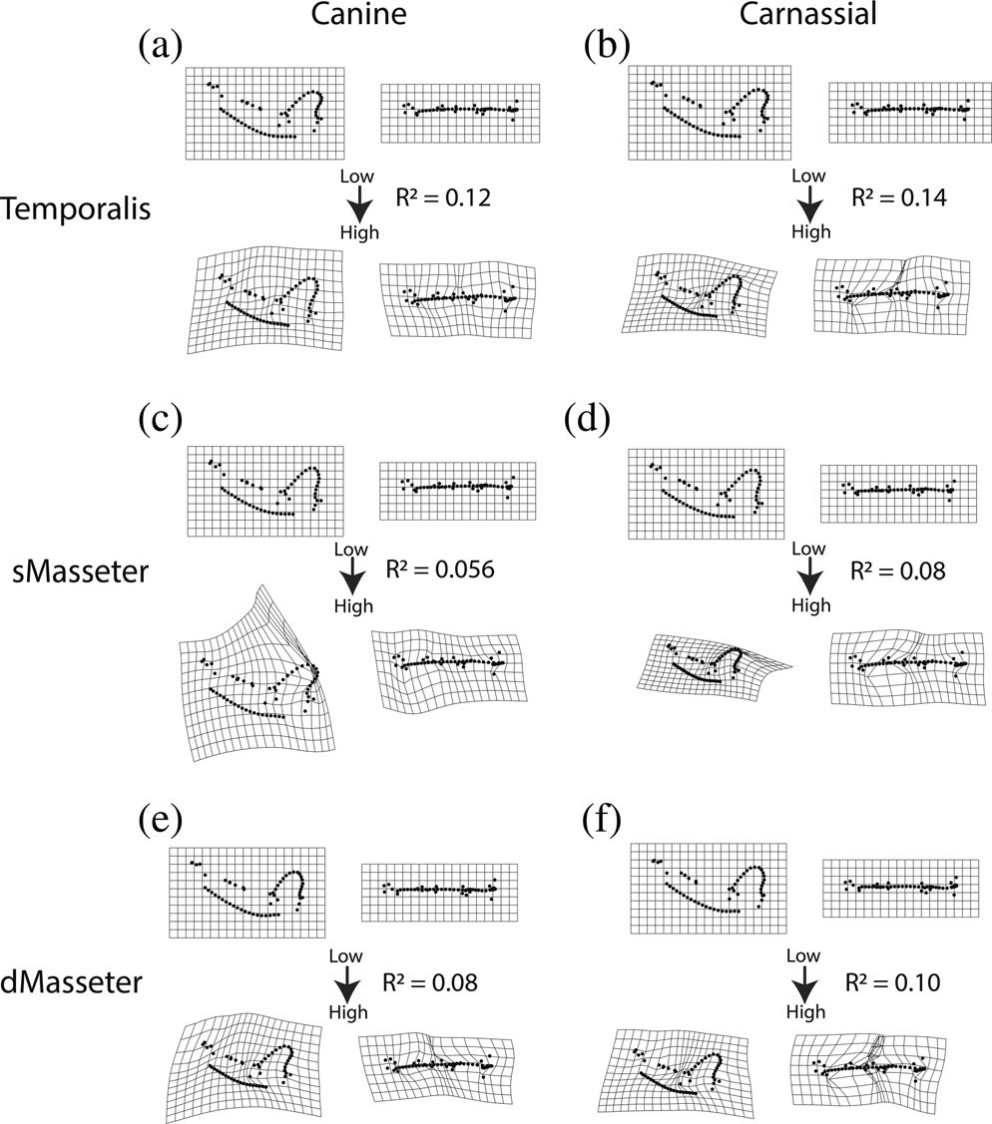
Spline deformations of mandible strength per mechanical advantage measurement with its associated *R* ^2^ value. (a) Mechanical advantage of the canine‐temporalis. (b) Mechanical advantage of the carnassial‐temporalis. (c) Mechanical advantage of the superficial masseter‐canine. (d) Mechanical advantage of the superficial masseter‐carnassial. (e) Mechanical advantage of the deep masseter‐canine. (f) Mechanical advantage of the deep masseter‐carnassial.

The deformation in shape associated with each of these functional parameters was visualized with thin plate splines, here shown as the shape associated with a high value of each metric (Figure 5). The functional parameter with the highest explanatory power, the mechanical advantage of the temporalis and the carnassial involved the shortening of the mandible, has a more vertical orientation of the mandibular symphysis, the reduction of the crushing area of the mandible, and the widening of the condyle (Figures 5b and S2). These characteristics are typically associated with greater degrees of hypercarnivory (e.g., reduction of crushing area of the mandible) and higher amounts of stress on the mandible (e.g., wider condyle, more vertical mandibular symphysis) but the lowest mechanical advantage value is from *Epicyon saevus* and the highest mechanical advantage value is from *Eoarctos*.

### 3.3 Functional morphology: finite element analysis

The permutation‐based analysis of variance (ANOVA) shows that stress and strain on the mandible are significantly different among mesocarnivores, hypocarnivores, hypercarnivores, and bone‐crackers within carnivores (three degrees of freedom and *p*‐values >0.01 for the canine load stress and strain and =0.02 for carnassial load stress and strain). On average, mesocarnivores have the highest stress and strain from both the canine load and the carnassial load (Table 3). Average stress and strains among hypocarnivores, hypercarnivores, and bone‐crackers are closer to one another, but hypercarnivores and bone‐crackers have lower average stress and strain from both the canine load and the carnassial load. In terms of differences in stress and strain between the different clades, “Miacidae” and Viverridae have the highest stress and strain from the canine load, followed by Amphicyonidae, Canidae, Procyonidae, Herpestidae, Ursidae, Mustelidae, Hyaenidae, and Felidae. “Miacidae” and Amphicyonidae have the highest stress and strain from the carnassial load, followed by Viverridae, Procyonidae, Ursidae, Canidae, Mustelidae, Felidae, Herpestidae, and Hyaenidae. Between the two suborders, caniforms have higher average stress and strain than feliforms.

**TABLE 3:** List of group means of the mesh‐weighted arithmetic mean Von Mises stresses and strains of the mandible from canine and carnassial loads with groups based on either ecology or clade.

Ecology	Canine stress (Pa)	Canine strain	Carnassial stress (Pa)	Carnassial strain
Mesocarnivore	1.58E+07	1.03E−03	4.02E+06	2.65E−04
Hypocarnivore	1.16E+07	7.56E−04	2.81E+06	1.83E−04
Hypercarnivore	1.08E+07	7.01E−04	2.48E+06	1.61E−04
Bone‐cracker	1.07E+07	6.98E−04	2.66E+06	1.73E−04
Family				
Viverridae	1.56E+07	1.01E−03	3.24E+06	2.11E−04
Miacidae	1.55E+07	1.00E−03	5.29E+06	3.56E−04
Amphicyonidae	1.40E+07	9.09E−04	3.84E+06	2.50E−04
Canidae	1.33E+07	8.65E−04	3.21E+06	2.09E−04
Procyonidae	1.33E+07	8.62E−04	3.29E+06	2.14E−04
Herpestidae	1.19E+07	7.72E−04	2.03E+06	1.32E−04
Ursidae	1.14E+07	7.44E−04	3.34E+06	2.17E−04
Mustelidae	1.07E+07	6.93E−04	2.36E+06	1.54E−04
Hyaenidae	1.03E+07	6.68E−04	1.87E+06	1.22E−04
Felidae	9.47E+06	6.15E−04	2.04E+06	1.33E−04

*Note*: List of group means of the MWAM Von Mises stress and strain of the mandible from canine and carnassial loads with groups based on ecology or clade. Stresses and strain were derived from scaled 100 N loads. It should be noted that Procyonidae and Herpestidae are both represented by 1 taxon.

When regressed onto Procrustes‐aligned shape data, stress and strain metrics are significantly nearly equal in their ability to explain the variation in mandible shape (Table 2). The stress and strain *R* ^2^ values are significant and comparable to the *R* ^2^ values of the mechanical advantage of the temporalis (Table 2). Stress and strain from canine loads were better at explaining shape than the carnassial loads. A MANCOVA of interactions between phylogeny or ecology increases the explanatory power. Similar to the mechanical advantage measurements, a PGLS showed decreases in *R* ^2^ values of the stress and strain measurements indicating an influence of phylogeny. Canine stress and strain had a significant (*p*‐value = 0.035) *R* ^2^ = 0.05, but carnassial stress and strain had insignificant (*p*‐value = 0.068) *R* ^2^ = 0.07 and 0.06, respectively. However, the canine stress and strain measurements are still comparable with the mechanical advantage measurements.

**FIGURE 6:**
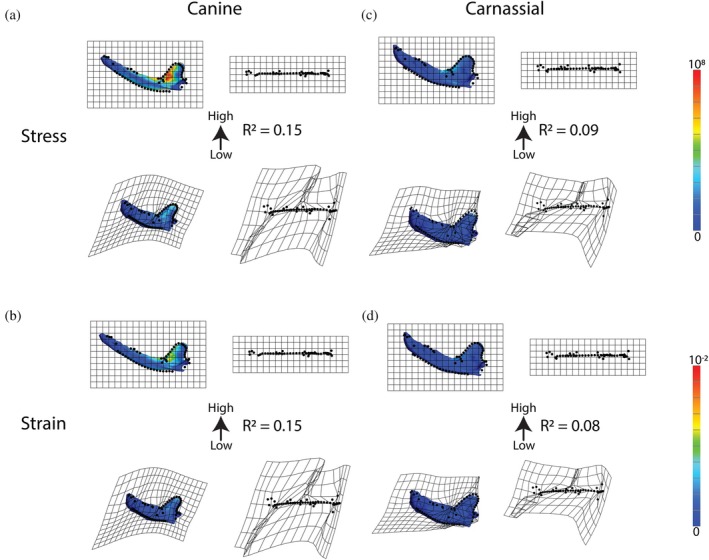
Spline deformations of mandible strength per stress and strain measurements from finite element analysis with its associated *R* ^2^ value and finite element model plot under the splines in the *XY* plane. Stress measurements are in pascals. (a) Canine stress. (b) Canine strain. (c) Carnassial stress. (d) Carnassial strain. High stress and strain finite element model plots are from the taxa *Vulpes* for the canine loads and *Amphicyon* for the carnassial load. Low stress and strain finite element model plots are from the taxon *Adcrocuta* for both the canine and carnassial loads.

As stress and strain on the mandible decreased, the mandible straightens and shortens, the crushing area of the molars is reduced, the condyle is more ventrally positioned and becomes wider, the canine increases in circumference, the mandibular symphysis increases in area and becomes more vertically oriented, and muscle attachment areas move more rostrally (Figure 6). *Adcrocuta*, a bone‐cracker of Hyaenidae, and *Pseudaelurus validus*, a hypercarnivore of Felidae, have the lowest stress and strain from both the canine and carnassial loads. *Vulpes* and *Miocyon*, mesocarnivores of Canidae and “Miacidae” respectively, have the highest stress and strain from the canine load, and *Amphicyon* and *Miocyon*, the former an Amphicyonidae, have the highest stress and strain from the carnassial load. *Miocyon*'s stress and strain values were noticeably high and may be due to errors in the repairs that were made to the original 3D model of the specimen.

## 4 DISCUSSION

### 4.1 Patterns of stress and strain on the mandible

Mesocarnivores tended to, on average, have higher stress and strain values from mandible loadings than specialized carnivore ecologies such as hypercarnivory and bone‐cracking hypercarnivory. The degree of carnivory appeared to be correlated with lower stress and strain except with hypocarnivores and carnassial loadings (Table 3) which tend to have lower stress and strain than hypercarnivores but not bone‐crackers. Mesocarnivores are adapted to a generalist diet with prey that is much smaller than them, with longer, often thinner mandibles in the dorsoventral direction, to catch prey that will not involve a lot of comparative stress on the mandible from struggling prey compared to a hypercarnivore (Christiansen, 2008; Christiansen & Adolfssen, 2005; Christiansen & Wroe, 2007; Davis, 2014; Martin, 1980; Meloro & O'Higgins, 2011; Roemer et al., 2009; Van Valkenburgh, 2007). In this study sample, the average mesocarnivore mandible shape included a greater curvature of the ventral side of the mandible, a thinner, more rostro‐caudal aligned mandibular symphysis, and a shorter coronoid process resulting in a smaller in‐lever arm (Figure 6). These are all adaptations meant to increase the reach of the mandible for prey capture and to rapidly close the jaws, but at the cost of robustness and strength of the mandible (Christiansen, 2008; Meloro et al., 2011; Piras et al., 2013; Prevosti et al., 2012; Van Valkenburgh, 2007).

**FIGURE 7:**
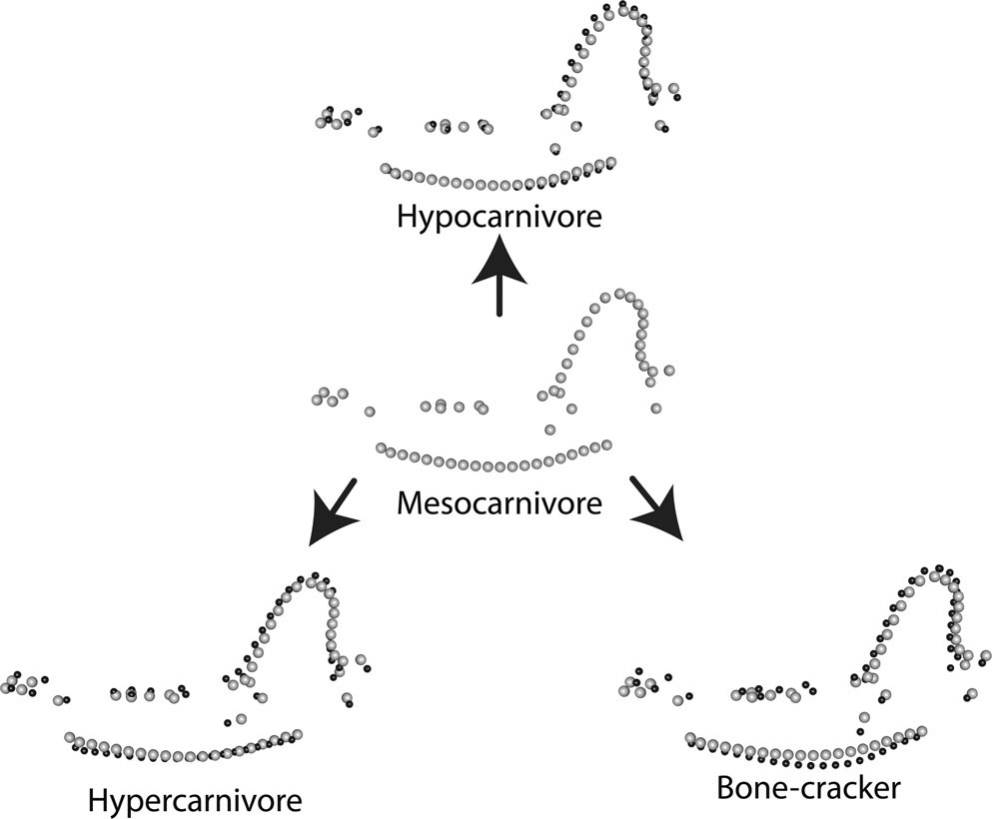
Point transformations of the mean mesocarnivore shape to either the mean hypocarnivore, mean hypercarnivore, or mean bone‐cracker shapes.

Hypercarnivores, including bone‐cracking hypercarnivores, had stronger mandibles with, on average, lower stress and strain than mesocarnivores. Being able to withstand high forces and reduce stress and strain is necessary for hypercarnivores to resist struggling prey that is equal to or larger than the carnivore in body size (Echarri et al., 2017; Meloro et al., 2011; Prevosti et al., 2012; Tseng et al., 2023; Tseng & Flynn, 2018). In this study, the average hypercarnivore mandible shape included a thicker, more dorsoventral‐aligned mandibular symphysis coupled with larger canines, a straighter ventral side of the mandible (more so with hypercarnivores) with only curving medially (the latter more pronounced in bone‐crackers), larger condyles, and a higher coronoid process and a more rostral (and for bone‐crackers more ventral) masseteric fossa for a longer in‐lever arm (Figure 7). An exception to this generalization is that bone‐crackers have a noticeable displacement between the position of the ascending ramus and the horizontal ramus of the mandible (Figure 7). The displacement between the two parts of the mandible increases the lever arm of the temporalis and displaces the condyle and the tooth row. The mandible shape produces a stronger bite force with the longer in‐lever arms from the temporalis and masseter muscles and a stronger, more resistant jaw to stronger bite forces and forces from struggling prey (a straighter and shorter horizontal ramus keeps mandible strength high at different parts of the mandible).

The shape of the mandibular symphysis and size of the canines are related to the strength of the mandible against struggling prey during prey capture. In hypercarnivores such as felids and some mustelids, large canines and a large mandibular symphysis add strength associated with capturing prey of equal to or larger size (Christiansen, 2008; Davis, 2014; Van Valkenburgh, 2007). The greater correlation of canine stress and strain on the mandible shape versus carnassial stress and strain emphasizes the importance of the canine area for hypercarnivory in mandible shape. It is possible that the correlation between the stress and strain metrics and mandible shape has more to do with relative prey size as opposed to diet.

**FIGURE 8:**
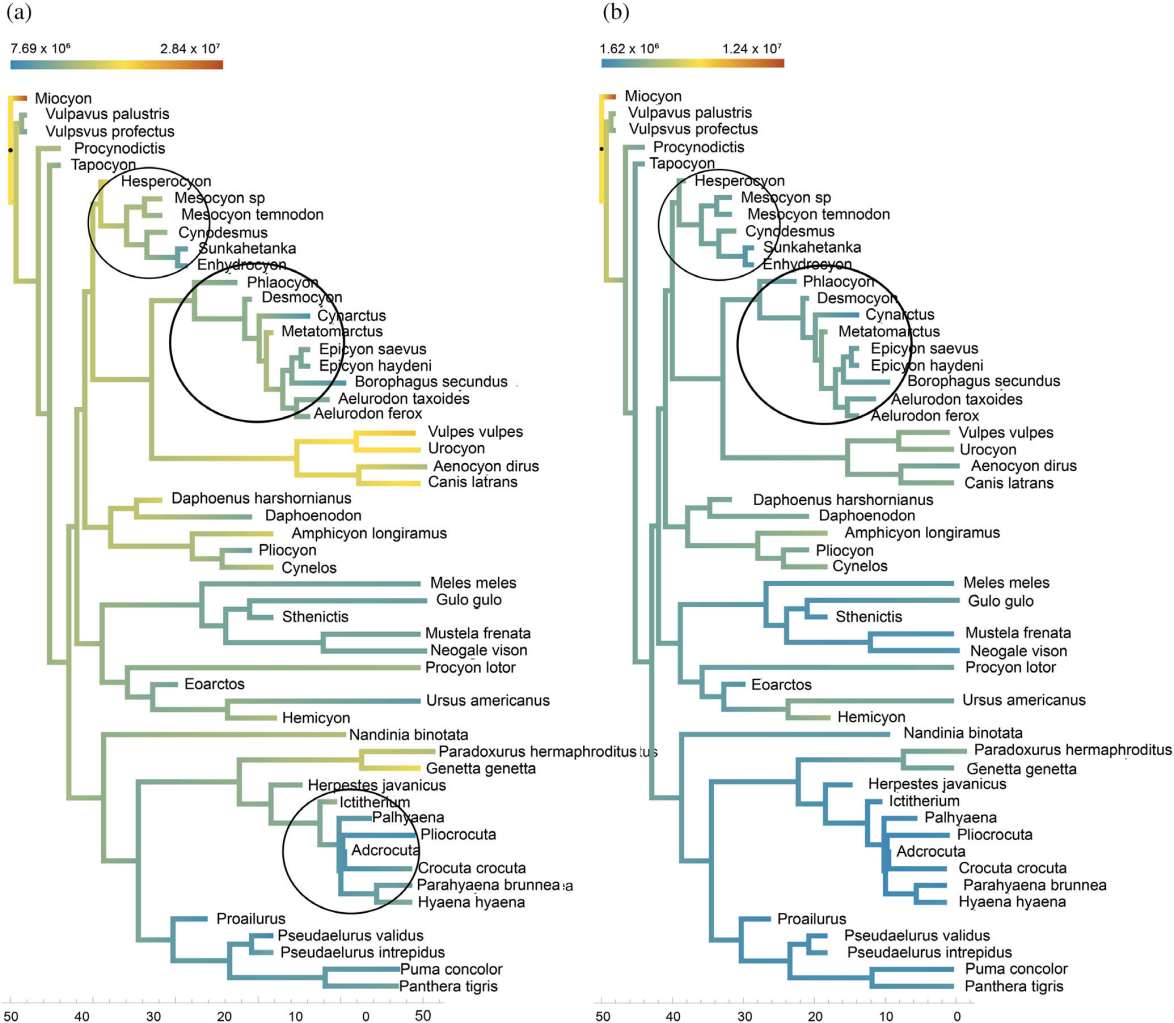
Changes in Von Mises stress on the mandibles of Carnivoraformes over time. (a) Canine stress. (b) Carnassial stress. The values of stress are in pascals (Pa). Circled areas highlight areas of the phylogenetic tree where noticeable changes in Von Mises stress occur in clades between basal mesocarnivorous taxa and derived hypercarnivores/bone‐cracking hypercarnivores. The scale on the bottom is time in millions of years. The Von Mises stress was mapped out via a standard ancestral state reconstruction under a Brownian motion of evolution (Felsenstein, 1973, 2004; Garland & Ives, 2000).

Looking at family groups, some patterns can be recognized between stress and strain and mesocarnivory versus hypercarnivory (Table 3). Groups that are generally mesocarnivorous, such as Viverridae, had higher stress and strain than groups that include hypercarnivores, such as Felidae. When the stresses are mapped onto a phylogenetic tree under a Brownian motion model of evolution, taxonomic groups that showed decreased canine stress and strain on the mandible as the degree of carnivory increases are mainly apparent in Canidae (*Hesperocyon* compared to terminal taxa of Hesperocyoninae and Borophaginae) and Hyaenidae (*Icitherium* vs. terminal taxa) (Figure 8a). An exception to the former was the members of the Caninae subfamily (i.e., *Vulpes*, *Urocyon*, *Canis latrans*, and *Aenocyon*) which are grouped together and are mostly mesocarnivores (although *Canis latrans*, the coyote, is considered a mesocarnivore, with the absence of *Canis lupus*, the gray wolf, or other hypercarnivores, it can become more hypercarnivorous (Avrin et al., 2023)). A pattern of repeated decreased stress and strain and evolution into carnivory in Canidae was clear because of the richness of the North American fossil record for Canidae (Balisi et al., 2018; Van Valkenburgh, 1991; Van Valkenburgh et al., 2004). The pattern of decreased stress and strain was harder to see in groups that rapidly went into hypercarnivory, such as Felidae (the basal member, *Proailurus*, being already a hypercarnivore and the only sister taxon to *Proailurus* in this study is *Nandinia*, a mesocarnivore/hypocarnivore of the Nandiniidae) (Christiansen, 2008; Hunt Jr, 1998) or groups that rapidly strengthened their mandibles (e.g., Hyaenidae in Figure 8b).

The trend of decreasing stress and strain from mesocarnivory to specialized carnivory was generally true for both canine and carnassial loads. An exception was Amphicyonidae, which had higher stress and strain measurements from the carnassial loads (Table 3). This may be due to amphicyonids having a more rostrally placed carnassial and larger postcarnassial molars. Upon placing the carnassial load onto the more caudal second molar, stress and strains on the mandible became more comparable to other bone‐crackers and hypercarnivores. Another exception was within Hyaenidae, as *Ictitherium* already experienced low stress and strain from the carnassial load. The exception with Hyaenidae could be a similar case to Felidae of a family rapidly acquiring the traits of a stronger mandible for the carnassial area.

### 4.2 Phylogeny versus ecology and their correlation with shape

The major axis of mandible shape (PC1) appeared to be correlated with ecology and function and generally separated mesocarnivores from hypercarnivores (Figure 4). There were instances of overlapping taxa between mesocarnivores and hypercarnivores/bone‐crackers across PC1, with notably *Amphicyon* and *Herpestes*. *Amphicyon*'s placement in morphospace and inability to be separated may be a testament to the difficulty in determining its ecology and diet in past literature (Sorkin, 2006). *Herpestes*'s placement might be phylogenetic, as it is often close to Viverridae (which are composed of mesocarnivores and hypocarnivores) which are the sister group of Herpestidae's larger taxonomic group, Herpestoidea, that it makes with Hyaenidae. Bone‐crackers had a noticeable overlap with other hypercarnivores, but bone‐crackers of similar shape and closer phylogeny tended to group around one another at combinations of PCs without PC1. Hypocarnivores also overlapped in a specific area with mesocarnivores, which might be attributed to aspects of the mandible shape that are related to the molar crushing area. However, hypocarnivores also overlap with bone‐crackers and hypercarnivores, which may be related to the larger area of mandible muscle attachment and mandible depth. Additionally, during regression of each functional metric onto the principal components, PC1 was consistently the most correlated with the functional metrics, as opposed to other PCs (Table 2).

Regression data showed that there was a strong correlation between shape and taxonomic grouping at the family level (*R* ^2^ = 0.38, *p*‐value = 0.001) but a weaker correlation at the suborder level (*R* ^2^ = 0.14, *p*‐value = 0.001). The PCA plots had shown distinct areas of morphospace occupied by distinct families such as Felidae and Hyaenidae (Figure 4b,d). However, caniform families tend to overlap with each other on the first PC axes of morphospace. The correlation between shape and family and what is seen from the PCA could mean that certain mandible morphologies evolved at the family level. Some of these morphologies could be related to instances of tooth row reduction and carnassial shape and placement. Prior studies have shown that at the family taxonomic level, carnivorans rapidly diversified into distinct tooth morphologies that persisted throughout the family (Hopkins et al., 2022). An exception for this lock‐in morphology may be North American carnivorans that evolved during the “cat‐gap” in the Miocene, such as *Enhydrocyon*, which has a more felid‐like skull and whose mandible plots closely with felids in the first PC axes (Figure 4). A similar ecological convergence in shape going beyond phylogenetic familial constraints may also be seen in the later borophagine canid species such as *Aelurodon*, *Epicyon*, and *Borophagus*, which have a more hyaenid‐like morphology in their mandibles.

## 5 CONCLUSION

While phylogeny is highly correlated with the variation of shape, the major axis of shape variation in carnivoran mandibles represented by the condyle position and width, carnassial blade length, length and curvature of the mandible, size of muscle attachment areas, and shape of the mandibular symphysis is noticeably correlated with functional ecology based on regression of functional metrics and the separation of mesocarnivores from more specialized hypercarnivores and bone‐crackers. Additionally, finite element analysis shows on average higher stress and strain on the mandible of mesocarnivores than on more specialized ecologies such as hypercarnivores and bone‐crackers.

Future work on this would include exploring if the patterns seen in this dataset are still observable in a two‐dimensional dataset. For the latter, it may be of interest to look at certain taxonomic groups that have a clearer trend of functional ecology changes at a family level (i.e., Canidae or Canidae specific subfamilies such as Hesperocyoninae and Borophaginae). Another point of interest is to look at how aspects of shape, functional performance, and dietary ecology change in tandem with one another (i.e., are they changing at similar rates in tandem with one another or is one of them changing faster than the others at a “lag”).

## AUTHOR CONTRIBUTIONS

**Charles J. Salcido:** Conceptualization; investigation; funding acquisition; writing – original draft; methodology; visualization; software; data curation; formal analysis. **P. David Polly:** Methodology; writing – review and editing; supervision.

## Supporting information


**FIGURE S1:** Principal component of shape for Carnivoraformes jaws for PC1 versus PC3. Grouping is based on either ecology (a) or phylogeny (b).

**FIGURE S2:** Principal components of shape for Carnivoraformes jaws that highlights differences in mechanical advantage of the temporals is the carnassial. Point colors are based on mechanical advantage values on a color gradient with low values in blue and high values in red. Splines are based on the ecology groupings.

**FIGURE S3:** Principle components of shape for Carnivoraformes jaws that highlight differences in all mechanical advantage measurements. Point colors are based on mechanical advantage values on a color gradient with low values in blue and high values in red. Splines are based on the ecology groupings.

**TABLE S1:** List of all measurements of specimens.
