# Supplementary figures and images for: The relationship between form and function of the carnivore mandible
Source: Anat Rec (Hoboken). 2025 Apr 30;309(9):2487–506. doi: 10.1002/ar.25678 (PMC13432026; doi:10.1002/ar.25678)

A.

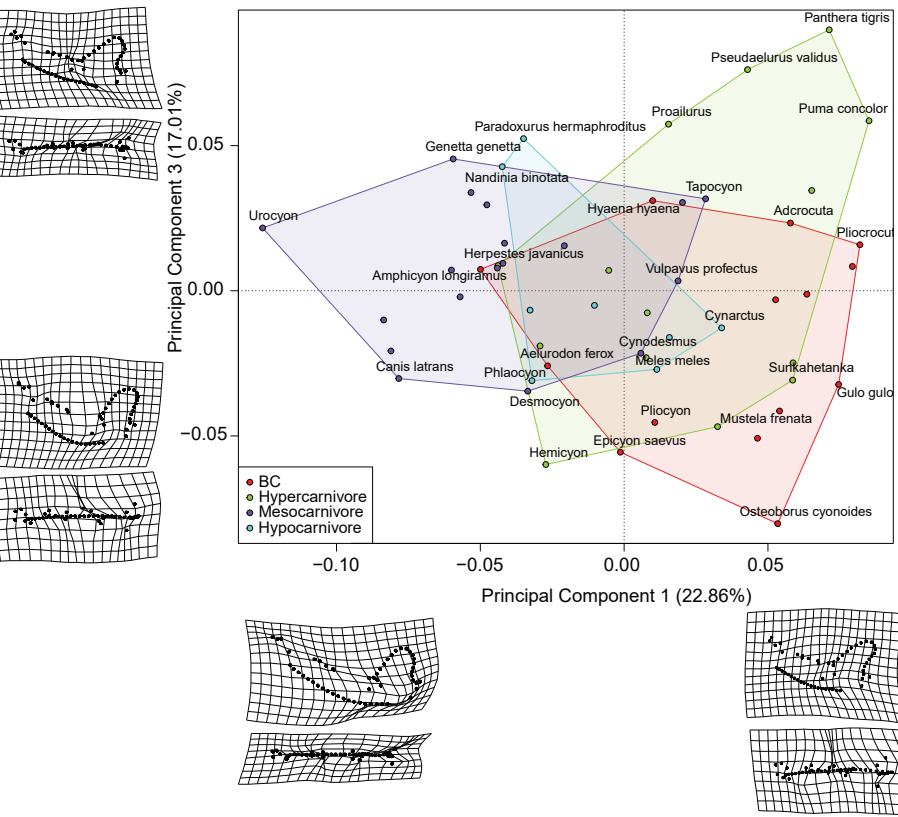

B.

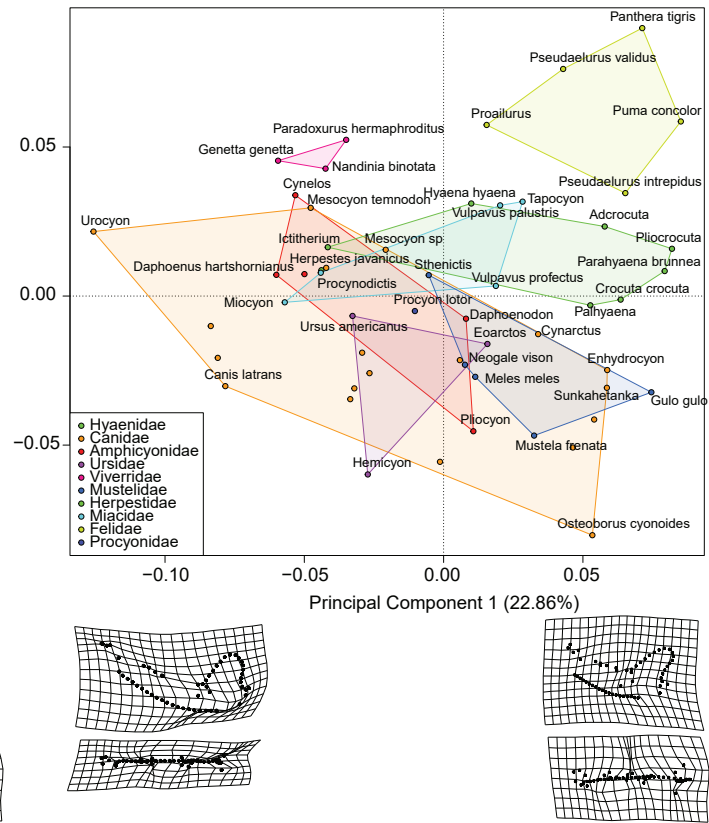

Supplement: Supplementary file 1 — FIGURE S1: Principal component of shape for Carnivoraformes jaws for PC1 versus PC3. Grouping is based on either ecology (a) or phylogeny (b). [file AR-309-2487-s004.pdf]
