# Supplementary material for: The relationship between form and function of the carnivore mandible
Source: Anat Rec (Hoboken). 2025 Apr 30;309(9):2487–506. doi: 10.1002/ar.25678 (PMC13432026; doi:10.1002/ar.25678)
Supplement: Supplementary file 3 — FIGURE S3: Principle components of shape for Carnivoraformes jaws that highlight differences in all mechanical advantage measurements. Point colors are based on mechanical advantage values on a color gradient with low values in blue and high values in red. Splines are based on the ecology groupings. [file AR-309-2487-s002.pdf]

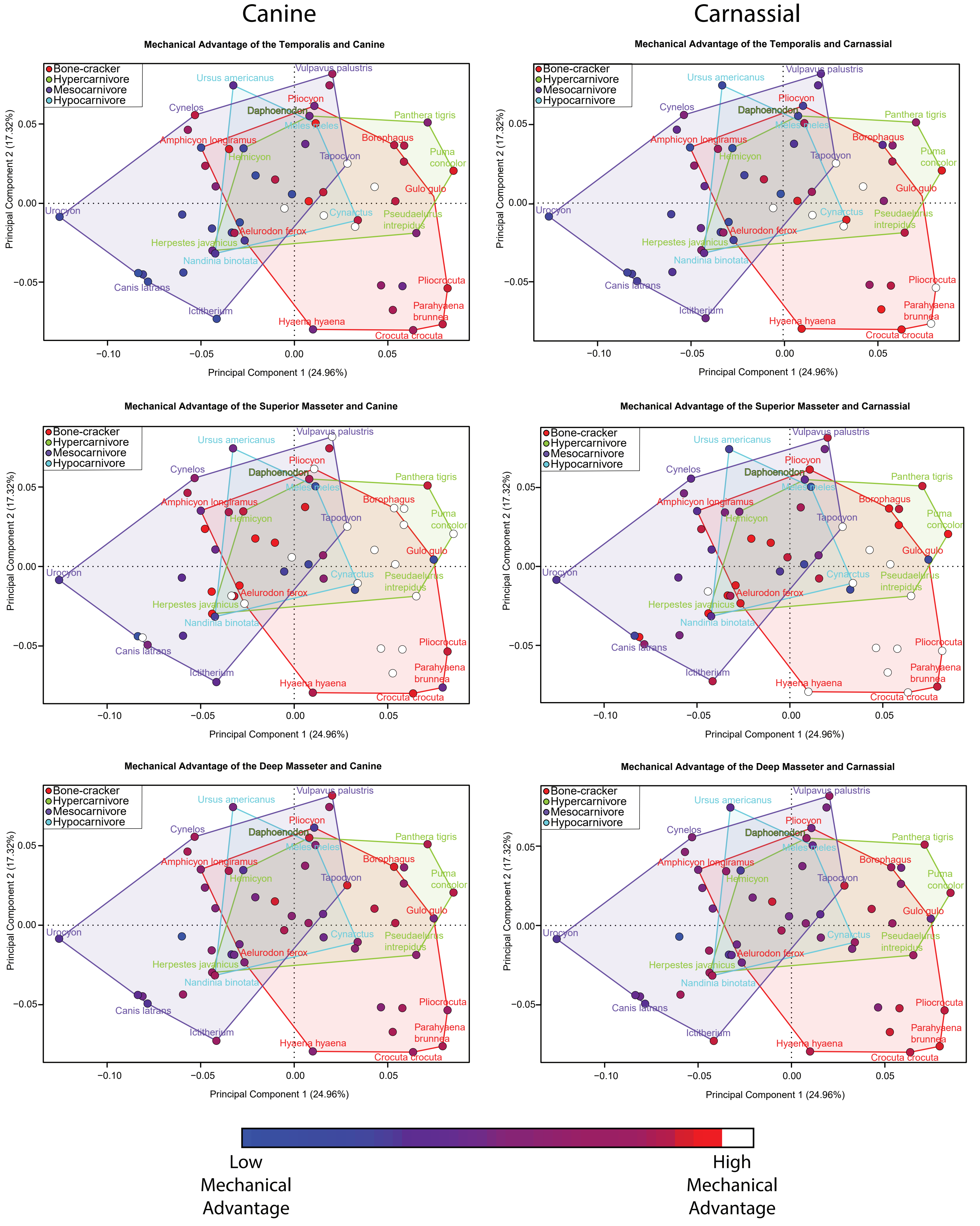
Canine
Carnassial
Mechanical Advantage of the Temporalis and Canine
Mechanical Advantage of the Temporalis and Carnassial
Mechanical Advantage of the Superior Masseter and Canine
Mechanical Advantage of the Superior Masseter and Carnassial
Mechanical Advantage of the Deep Masseter and Canine
Mechanical Advantage of the Deep Masseter and Carnassial
Bone-cracker
Hypercarnivore
Mesocarnivore
Hypocarnivore
Vulpavus palustris
Ursus americanus
Pliocyon
Cynelos
Daphoenodon
Panthera tigris
Meles meles
Amphicyon longiramus
Borophagus
Hemicyon
Puma concolor
Tapocyon
Gulo gulo
Urocyon
Cynarctus
Pseudaelurus intrepidus
Aelurodon ferox
Herpestes javanicus
Nandinia binotata
Pliocrocuta
Canis latrans
Parahyaena brunnea
Ictitherium
Hyaena hyaena
Crocuta crocuta
Principal Component 1 (24.96%)
Principal Component 2 (17.32%)
−0.10
−0.05
0.00
0.05
Low Mechanical Advantage
High Mechanical Advantage
